# Generation of two induced pluripotent stem cell lines and the corresponding isogenic controls from Parkinson’s disease patients carrying the heterozygous mutations c.1290A > G (p.T351A) or c.2067A > G (p.T610A) in the RHOT1 gene encoding Miro1

**DOI:** 10.1016/j.scr.2023.103085

**Published:** 2023-06

**Authors:** Axel Chemla, Giuseppe Arena, Claudia Saraiva, Clara Berenguer-Escuder, Dajana Grossmann, Anne Grünewald, Christine Klein, Philip Seibler, Jens C. Schwamborn, Rejko Krüger

**Affiliations:** a Translational Neuroscience, Luxembourg Centre for Systems Biomedicine (LCSB), University of Luxembourg, Luxembourg; b Developmental and Cellular Biology, Luxembourg Centre for Systems Biomedicine (LCSB), University of Luxembourg, Luxembourg; c Translational Neurodegeneration Section “Albrecht-Kossel”, Department of Neurology, University Medical Center Rostock, University of Rostock, Rostock, Germany; d Institute of Neurogenetics, University of Lübeck, Lübeck, Germany; e Molecular and Functional Neurobiology, Luxembourg Centre for Systems Biomedicine (LCSB), University of Luxembourg, Luxembourg; f Transversal Translational Medicine, Luxembourg Institute of Health (LIH), Luxembourg; g Parkinson Research Clinic, Centre Hospitalier de Luxembourg (CHL), Luxembourg

## Abstract

Primary skin fibroblasts from two Parkinson’s disease (PD) patients carrying distinct heterozygous mutations in the *RHOT1* gene encoding Miro1, namely c.1290A > G (Miro1 p.T351A) and c.2067A > G (Miro1 p.T610A), were converted into induced pluripotent stem cells (iPSCs) by episomal reprogramming. The corresponding isogenic gene-corrected lines have been generated using CRISPR/Cas9 technology. Here, we provide a comprehensive characterization and quality assurance of both isogenic pairs, which will be used to study Miro1-related molecular mechanisms underlying neurodegeneration in iPSC-derived neuronal models (e.g., midbrain dopaminergic neurons and astrocytes).

## 1 Resource table

**Table:** 

Unique stem cell line identifier	1. LCSBi011A 2. LCSBi011A-1 3. LCSBi012A 4. LCSB012A-1
Alternative name(s) of stem cell line	1. RHOT1_T351A_clone1_PD 2. RHOT1_T351A_clone25.2_IsogenicControl 3. RHOT1_T610A_clone6_PD 4. RHOT1_T610A_clone62.19.37_IsogenicControl
Institution	*Luxembourg Centre for Systems Biomedicine (LCSB), University of Luxembourg, Luxembourg*
Contact information of the reported cell line distributor	*Prof. Rejko Krüger; rejko.krueger@uni.lu*
Type of cell line	*iPSCs*
Origin	*Human*
Additional origin info *(applicable for human ESC or iPSC)*	1. Age at biopsy: 65 years *Sex: male Ethnicity: European White* 2. As in 1. 3. Age at biopsy: 45 years *Sex: male Ethnicity: European White* 4. As in 3.
Cell Source	*Dermal fibroblasts*
Method of reprogramming	*Electroporation of episomal reprogramming vectors*
Clonality	*Clonal*
Evidence of the reprogramming transgene loss (including genomic copy if applicable)	*Loss of reprogramming plasmids was confirmed by PCR*
The cell culture system used	*iPSCs were maintained under feeder-free conditions, on Matrigel-coated wells, in presence of Essential 8™ (E8) medium*
Type of the Genetic Modification	*Spontaneous point mutation (LCSBi011A and LCSBi012A)* *Gene correction (LCSBi011A-1 and LCSB012A-1)*
Associated disease	*Parkinson disease (OMIM #168600)*
Gene/locus	*RHOT1 (17q11.2), Gene ID: 55288, NM_001033568.2*
Method of modification/user-customisable nuclease (UCN) used, the resource used for design optimisation	*CRISPR/Cas9*
User-customisable nuclease (UCN) delivery method	*Electroporation*
All double-stranded DNA genetic material molecules introduced into the cells	*Episomal reprogramming vectors, sgRNA plasmids, repair template plasmid*
Analysis of the nuclease-targeted allele status	*Sanger Sequencing of the targeted alleles*
Method of the off-target nuclease activity prediction and surveillance	*The “CRISPR-Cas9 guide RNA design checker” tool (from Integrated DNA Technology, Inc.) was used to assess on– and off-target potential of selected* guide RNA *sequences*
Descriptive name of the transgene	*N/A*
Eukaryotic selective agent resistance cassettes (including inducible, gene/cell type-specific)	*N/A*
Inducible/constitutive expression system details	*N/A*
Date archived/stock creation date	*01.12.2022*
Cell line repository/bank	*https://hpscreg.eu/cell-line/LCSBi011-A* *https://hpscreg.eu/cell-line/LCSBi011-A-1* *https://hpscreg.eu/cell-line/LCSBi012-A* *https://hpscreg.eu/cell-line/LCSBi012-A-1*
Ethical/GMO work approvals	*The Luxembourgish National Research Ethics Committee (CNER) provided ethical approval for the following project: “Disease modelling of Parkinson’s disease using patient-derived fibroblasts and induced pluripotent stem cells” (DiMo-PD, CNER #201411/05).*
Addgene/public access repository recombinant DNA sources’ disclaimers (if applicable)	*pEP4 E02S ET2K (Addgene #20927)* *pEP4 E02S EN2L (Addgene #20922)* *pEP4 E02S EM2K (Addgene #20923)* *pSimple-miR302/367 (Addgene #98748)* *pSMART-sgRNA (Sp) plasmid (Addgene #80427)* *pSMART HC Kan (Addgene #178883)*

## 2 Resource utility

We recently provided evidence supporting the involvement of heterozygous mutations in the *RHOT1* gene encoding Miro1 in PD pathogenesis (Berenguer-Escuder et al., 2019, Berenguer-Escuder et al., 2020, Grossmann et al., 2019, Grossmann et al., 2020). Here, we reported generation of iPSC lines from PD patients carrying two distinct *RHOT1* mutations that will be used, together with the corresponding gene-corrected lines, for phenotypic analysis and *in vitro* disease modeling.

## 3 Resource details

**Fig. 1:**
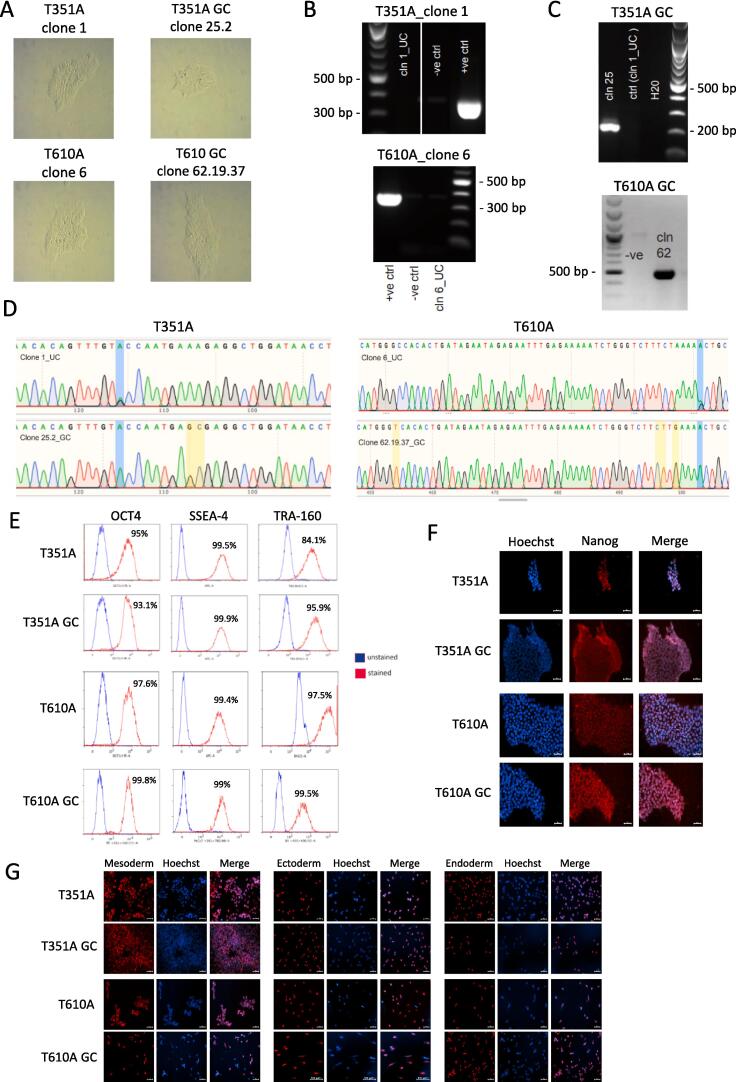

To generate the parental iPSC lines (i.e., *LCSBi011A* and *LCSBi012A*), primary skin fibroblasts were obtained from two PD patients carrying the *RHOT1* heterozygous mutations c.1290A > G (Miro1 p.T351A) and c.2067A > G (Miro1 p.T610A), which are located in the second EF-hand (EF2) and in the transmembrane (TMD) protein domain, respectively (Berenguer-Escuder et al., 2019). Clonal iPSC populations were derived following electroporation of episomal reprogramming vectors into patient fibroblasts. Individual iPSC colonies (clone 1 for Miro1 p.T351A, clone 6 for Miro1 p.T610A) were identified by morphology, isolated and expanded (Fig. 1A, left panels). Loss of reprogramming vectors was confirmed by PCR analysis using primers specific to the AmpR gene (Fig. 1B). Isogenic controls (i.e., *LCSBi011A-1 and LCSBi012A-1)* were generated by introducing gene editing factors into the aforementioned iPSC clones. Briefly, iPSCs were co-transfected with the specific sgRNA (Table 2), the Cas9 mRNA and a repair template comprising homology arms flanking the target site (Table 2, Supplementary Figs. 1 and 2). Gene-edited iPSCs were identified by PCR using allele-specific primers flanking the intended correction (Fig. 1C). The resulting amplicons were Sanger sequenced to confirm the successful gene editing and the absence of indel mutations. For the Miro1 p.T351A gene correction, a single iPSC colony (clone 25) showing a substantial proportion of edited cells was subjected to subcloning to obtain a pure culture of gene-corrected cells. Individual colonies were again isolated, expanded and screened by PCR and Sanger sequencing. One subclone (clone 25.2) showed the complete loss of the c.1290A > G mutation and gain of the synonymous changes (Fig. 1D, left panel). For the gene editing of Miro1 p.T610A, the sequencing analysis initially revealed not only the successful correction of the c.2067A > G mutation, but also the presence of an indel mutation in the wild-type allele of all clones screened, namely a 1 bp insertion (T) at the expected Cas9 cutting site. We therefore selected a single c.2067A > G gene-corrected colony (clone 62) and performed a second round of gene editing to correct the insertion in the second allele. The transfection was realized as described above, but using an alternative sgRNA that specifically recognizes the insertion mutation (Table 2). A colony (clone 62.19) containing a significant proportion of corrected iPSCs (identified by PCR) was further selected for subcloning to attain a pure population of gene-edited cells. Individual colonies were again isolated, expanded and screened. One subclone (clone 62.19.37) showed complete loss of both, the Miro1 c.2067A > G mutation and the 1 bp insertion, along with homozygous gain of the synonymous changes (Fig. 1D, right panel). Miro1 T351A_25.2 and T610A_62.19.37 corrected clones both displayed a typical iPSC colony morphology (Fig. 1A, right panels). Patient-derived and gene-edited clones were then expanded and subjected to further characterization. They all expressed different stemness markers, as demonstrated by both FACS analysis (Fig. 1E) and immunofluorescence (Fig. 1F). Pluripotency capacity was also confirmed by their ability to differentiate into the three germ layers (Fig. 1G). SNP array analysis excluded the presence of aneuploidies in all clones (Supplementary Fig. 3). Genetic identity between patient-derived iPSCs and the corresponding gene-edited clones was confirmed by SNPduo comparative assessment (Supplementary Fig. 4). Finally, all clones were free from mycoplasma contamination (Supplementary Fig. 5).

**Table 1:** Characterization and validation.

Classification (optional italicized)	Test	Result	Data
Morphology	Photography	*Typical iPSC morphology*	Fig. 1*panel A*
Pluripotency status evidence for the described cell line	Qualitative analysis *(*i.e. *Immunocytochemistry, western blotting)*	*All clones display a robust nuclear staining of the stemness marker Nanog*	Fig. 1*panel F*
	Flow cytometry	*All clones are positive for the pluripotency markers OCT4, SSEA-4 and Tra1-60*	Fig. 1*panel E*
Karyotype	SNP array Illumina Infinium GSA-24 v3.0, 0.50 Mb	*No aneuploidies detected* *Arr(X,Y)x1,(1*–*22)x2 for all clones*	*Supplementary Fig. 3*
Genotyping for the desired genomic alteration/allelic status of the gene of interest	PCR across the edited site or targeted allele-specific PCR	*Successful correction of the intended mutations*	Fig. 1*panel C*
	Evaluation of the – (homo-/hetero-/hemi-) zygous status of introduced genomic alteration(s)	*N/A*	*N/A*
	Transgene-specific PCR (when applicable)	*N/A*	*N/A*
Verification of the absence of random plasmid integration events	PCR	*Loss of reprogramming vectors confirmed*	Fig. 1*panel B*
Parental and modified cell line genetic identity evidence	SNPduo analysis of SNP array	*Identical genotypes between parental and gene edited lines*	*Supplementary Fig. 4*
		
Mutagenesis/genetic modification outcome analysis	Sanger Sequencing	*Confirmation of the homozygous corrections of the intended mutations*	Fig. 1*panel D*
	PCR-based analyses	*N/A*	*N/A*
	Southern Blot or WGS; western blotting (for knock-outs, KOs)	*N/A*	*N/A*
Off-target nuclease activity analysis	PCR across top 5/10 predicted top likely off-target sites, whole genome/exome sequencing*[*Optional but highly-recommended if Cas editing is used*]*	*N/A*	*N/A*
Specific pathogen-free status	Mycoplasma	*Negative for mycoplasma contamination*	*Supplementary Fig. 5*
Multilineage differentiation potential	Directed differentiation	*Ability of all clones to differentiate into the three germ layers*	Fig. 1*panel G*
*Donor screening (OPTIONAL)*	HIV 1 + 2 Hepatitis B, Hepatitis C	*N/A*	*N/A*
*Genotype* – *additional histocompatibility info (OPTIONAL)*	Blood group genotyping	*N/A*	*N/A*
	HLA tissue typing	*N/A*	*N/A*

**Table 2:** Reagent details.

Antibodies and stains used for immunocytochemistry/flow-cytometry			
	Antibody	Dilution	Company Cat # and RRID
*Pluripotency Markers (FACS)*	*TRA-1*–*60 BV421*	*As per manufacturer’s specification*	*BD Biosciences, Cat No. 562711; RRID: AB_2737738*
*Pluripotency Markers (FACS)*	*SSEA4 AF647*		*BD Biosciences, Cat No. 330408; RRID: AB_1089200*
*Pluripotency Markers (FACS)*	*OCT3/4 PE*		*BD Biosciences, Cat No. 560186; RRID: AB_1645331*
*Pluripotency Markers (ICC)*	*Rabbit anti Nanog*	*1:500*	*Abcam, Cat #: ab21624; RRID: AB_446437*
*Secondary antibody*	*Alexa Fluor 568 Goat anti-Rabbit IgG (H + L)*	*1:1000*	*Invitrogen, Cat #: A11036; RRID: AB_143011*
*Mesoderm Marker*	*Goat anti Brachyury*	*1:500*	*Human Pluripotent Stem Cell Functional Identification Kit (R&D Systems, Cat No. SC027B)*
*Endoderm Marker*	*Goat anti SOX-17*	*1:500*	
*Ectoderm Marker*	*Goat anti OTX2*	*1:500*	
*Secondary antibody*	*Alexa Fluor 647 Donkey anti Goat IgG (H + L)*	*1:1000*	*Invitrogen, Cat No. A21447; RRID: AB_141844*

Site-specific nuclease			
*Nuclease information*	*Cas9-geminin*	*In vitro transcribed mRNA*	
*Delivery method*	*Electroporation*	*Neon transfection system (1100 V, 30 ms, 1 pulse)*	
*Selection/enrichment strategy*	*Any*	*Any*	

Primers and Oligonucleotides used in this study			
	Target	Forward/Reverse primer (5′-3′)	
*Primers for verification loss of reprogramming vectors*	*AmpR cassette*	*CAGTCTATTAATTGTTGCCGGG*/*GCTATGTGGCGCGGTATTAT*	
*sgRNA Miro1 c.1290A > G mutation*	*RHOT1 exon 13*	*CAGTTTGTGCCAATGAAAGA*	
*sgRNA Miro1 c.2067A > G mutation*	*RHOT1 exon 19*	*AATTCTCTATTCTATCAGTG*	
*Miro1 c.1290A > G ssODN repair template*	*RHOT1 exon 13*	*GCCAGATGTGAATAACACAGTTTGTACCAATGAGCGAGGCTGGATAACCTACCAGGGATTCCTT*	
*Miro1 c.2067A > G repair template (plasmid)*	*RHOT1 exon 19*	*AGACATGGGTCACACTGATAGAATAGAGAATTTGAGAAAAATCTGGGTCTTCTTGAAAACTGCT*	
*sgRNA for correction indel mutation iPSCs Miro1 c.2067A > G GC*	*1 bp insertion (T) at the Cas9 cut site*	*ATTCTCTATTCTATCAAGTG*	
*Screening PCR* *Miro1 c.1290A > G* *gene-corrected clones*	*RHOT1 exon 13*	*CACAGTTTGTACCAATGAGC*/*CATCTTAGAGATATCAGCAGC*	
*Screening PCR* *Miro1 c.2067A > G* *gene-corrected clones*	*RHOT1 exon 19*	*GAGAAAAATCTGGGTCTTCTTG*/*GCTACTAAGTCTCTGCCAGC*	
*Sanger sequencing Miro1 c.1290A > G gene-corrected clones*	*RHOT1 exon 13*	*GCTTTGTCACCTGATGAGC*/*CATCTTAGAGATATCAGCAGC*	
*Sanger sequencing Miro1 c.2067A > G gene-corrected clones*	*RHOT1 exon 19*	*CAAGCACATAACTGTGGTCATC*/*GCTACTAAGTCTCTGCCAGC*	

## 4 Materials and methods

### 4.1 Cell culture and reprogramming

PD patient-derived fibroblasts were grown in DMEM medium containing 4.5 g/L D-glucose, 10% FBS and 1% Pen/Strep (Thermo Fisher Scientific, Braunschweig, Germany) at 37 °C 5% CO_2_. For iPSC generation, episomal vectors expressing reprogramming factors were introduced into fibroblasts by electroporation, using the Neon transfection system (1400 V, 20 ms, 2 pulses). Transfected cells were plated on Matrigel-coated wells in Essential 8 (E8) medium (Thermo Fisher Scientific) supplemented with 10 µM ROCK inhibitor, Y-27632 (Tocris). Medium changes were performed every day (without Y-27632) and individual iPSC colonies were isolated and expanded in E8 medium. Newly established iPSC lines were maintained in E8 medium (daily feeding) and passaged (1:4–1:6) every 3–4 days with 0.5 mM EDTA in PBS (Thermo Fisher Scientific). Bright field images confirmed the typical iPSC colony morphology for all lines (Fig. 1A).

### 4.2 Reprogramming vector analysis

Loss of reprogramming vectors was confirmed by PCR using primers specific to the AmpR gene (Table 2), which is present in all plasmids encoding reprogramming factors. Briefly, 250 ng of gDNA from each iPSC clone (after >5 passages) was used in the PCR analysis (performed over 30 cycles). gDNA extracted from vector-free iPSCs was used as a negative control (-ve ctrl), whereas 1 ng of reprogramming plasmid was used as a positive control (+ve ctrl). An expected band of 350 bp was observed for both Miro1 p.T351A (cln 1_UC) and Miro1 p.T610A (cln 6_UC) uncorrected clones (Fig. 1B). Ladder: 1 kb plus DNA ladder (NEB).

### 4.3 Gene editing

Isogenic lines were generated by CRISPR/Cas9-mediated gene editing (Supplementary Figs. 1 and 2). Briefly, iPSCs were co-transfected with the Cas9-geminin mRNA, the pSMART-sgRNA (Sp) plasmid (encoding a guide RNA specific for either Miro1 p.T351A or Miro1 p.T610A) and a repair template incorporating the mutation and synonymous base changes that facilitate PCR screening and prevent Cas9-mediated re-cutting (Table 2). The repair template consisted of a single-stranded oligodeoxynucleotide (ssODN) for the correction of Miro1 p.T351A (Supplementary Fig. 1, Table 2), whereas it was cloned into the minimal pSMART HC Kan plasmid for the correction of Miro1 p.T610A (Supplementary Fig. 2, Table 2). The “CRISPR-Cas9 guide RNA design checker” tool (from Integrated DNA Technology, Inc.) was used to assess on– and off-target potential of the selected guide RNA sequences. Gene-editing factors were introduced into a single iPSC clone using the Neon transfection system (1100 V, 30 ms, 1 pulse). Electroporated cells were plated on Matrigel-coated wells in E8 medium supplemented with 10 µM ROCK inhibitor. The next day, the medium was switched to E8 without ROCK inhibitor, and changed every other day. Individual colonies were isolated and expanded in E8 medium. Subcloning was performed by dissociating the cells with TryPLE (Thermo Fisher Scientific), followed by plating at low density in E8 medium supplemented with Y-27632 (which was removed after 24 h).

### 4.4 Allele-specific PCR and Sanger sequencing

gDNA was extracted using the DNeasy Blood and Tissue kit (Qiagen) according to the manufacturer’s instructions. Gene-edited iPSC clones were identified by PCR using specific primers recognizing the corrected alleles (Table 2). Expected bands of 213 bp and 502 bp respectively, were observed for Miro1 p.T351A (cln 25_GC) and Miro1 p.T610A (cln 62_GC) gene-corrected clones (Fig. 1C). Uncorrected Miro1 p.T351A (cln1_UC) and Miro1 p.T610A (cln1_UC, -ve) iPSC clones were used as negative controls (Fig. 1C). PCR amplicons generated with primers flanking the intended correction (Table 2) were Sanger sequenced to confirm the successful gene editing and the absence of indel mutations. Location of the uncorrected and gene-corrected mutations is highlighted in blue; homozygous incorporations of synonymous changes in the gene-edited clones are highlighted in yellow (Fig. 1D).

### 4.5 Pluripotency assays

Expression of stemness markers was evaluated by both FACS analysis and immunocytochemistry (ICC). For FACS, iPSCs were harvested with TrypLE and stained with the following antibodies: TRA-1-60 BV421 and SSEA4-AF647 were incubated in PBS + 2% FBS; OCT3/4 PE staining was performed on cells pre-treated with the Foxp3 fixation/permeabilisation buffer (Thermo Fisher Scientific, Cat No. 00-5523-00). Cells were analyzed using a FACSaria x-20 flow cytometer, with unstained iPSCs used as a gating control (Fig. 1E).

For the ICC, iPSCs were plated on Matrigel-coated coverslips and fixed in PBS + 4% PFA for 15 min. Cells were then permeabilized and blocked for 1 h in PBS supplemented with 10% goat serum, 2% BSA and 0.4% Triton-X 100, followed by overnight incubation at 4 °C with the Nanog antibody (Table 2), diluted in 1% goat serum, 0.2% BSA and 0.1% Triton-X 100. The following day, cells were washed in PBS and incubated with the corresponding secondary antibody for 1 h (Table 2). Finally, nuclei were stained with Hoechst and images acquired using a Zeiss AxioObserverZ1 microscope (Carl Zeiss Microimaging GmBH). Scale bar = 50 µm (Fig. 1F).

### 4.6 Three germ layer differentiation

iPSCs were plated on Matrigel-coated coverslips two days before the *in vitro* differentiation procedure started. The Human Pluripotent Stem Cell Functional Identification Kit (R&D Systems, Cat No. SC027B) was used to verify iPSCs capacity to differentiate into the three germ layers. Immunocytochemistry of the ectodermal marker OTX2, the mesodermal marker Brachyury and the endodermal marker SOX17 was performed. Images were acquired using a Zeiss AxioObserverZ1 microscope, scale bar = 50 µm (Fig. 1G).

### 4.7 Karyotyping and genetic identity

Genomic DNA was isolated from iPSCs and analyzed (Victorian Clinical Genetics Service, Murdoch Children’s Research Institute, Australia) using an Illumina Infinium CoreExome-24 v1.1 SNP array (Supplementary Fig. 3). Genetic identity between gene-edited and parental lines was assessed by SNPduo analysis (https://pevsnerlab.kennedykrieger.org/SNPduo/) of the SNP array (Supplementary Fig. 4).

### 4.8 Mycoplasma analysis

iPSCs were submitted to Cerberus Sciences (https://www.cerberus.net.au/submission) for mycoplasma testing and confirmed all negative (Supplementary Fig. 5).

## Declaration of Competing Interest

The authors declare that they have no known competing financial interests or personal relationships that could have appeared to influence the work reported in this paper.
