# Supplementary material for: Generation of two induced pluripotent stem cell lines and the corresponding isogenic controls from Parkinson’s disease patients carrying the heterozygous mutations c.1290A > G (p.T351A) or c.2067A > G (p.T610A) in the RHOT1 gene encoding Miro1
Source: Stem Cell Res. 2023 Jun;69:103085. doi: 10.1016/j.scr.2023.103085 (PMC10240566; doi:10.1016/j.scr.2023.103085)
Supplement: Supplementary data 1 [file mmc1.docx]

| 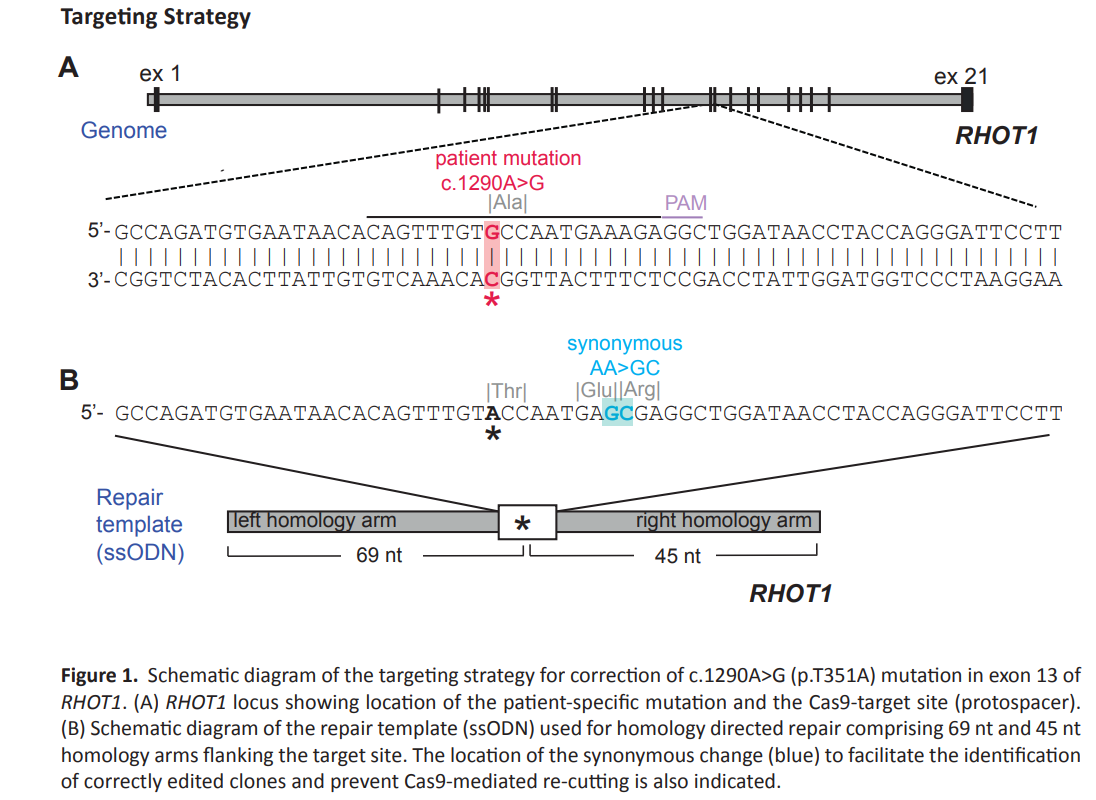 |
| --- |
| 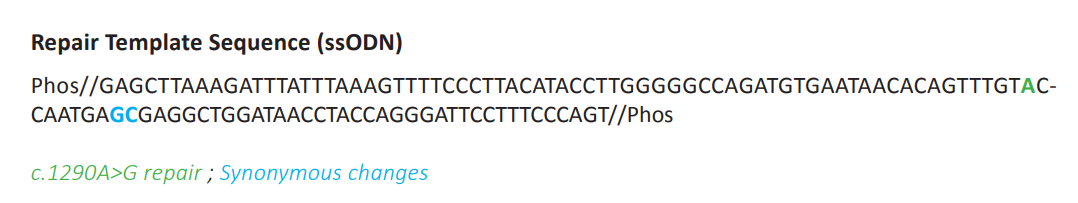 |
| **Supplementary Figure 1:** Schematic diagram of the targeting strategy for correction of c.1290A>G (p.T351A) mutation in exon 13 of RHOT1. (A) RHOT1 locus showing location of the patient-specific mutation (highlighted in red) and the Cas9-target site (protospacer). (B) Schematic diagram of the repair template (ssODN) used for homology directed repair comprising 69 nt and 45 nt homology arms flanking the target site. Synonymous changes (highlighted in blue) have been introduced to facilitate the identification of correctly edited clones and to prevent Cas9-mediated re-cutting. |

| 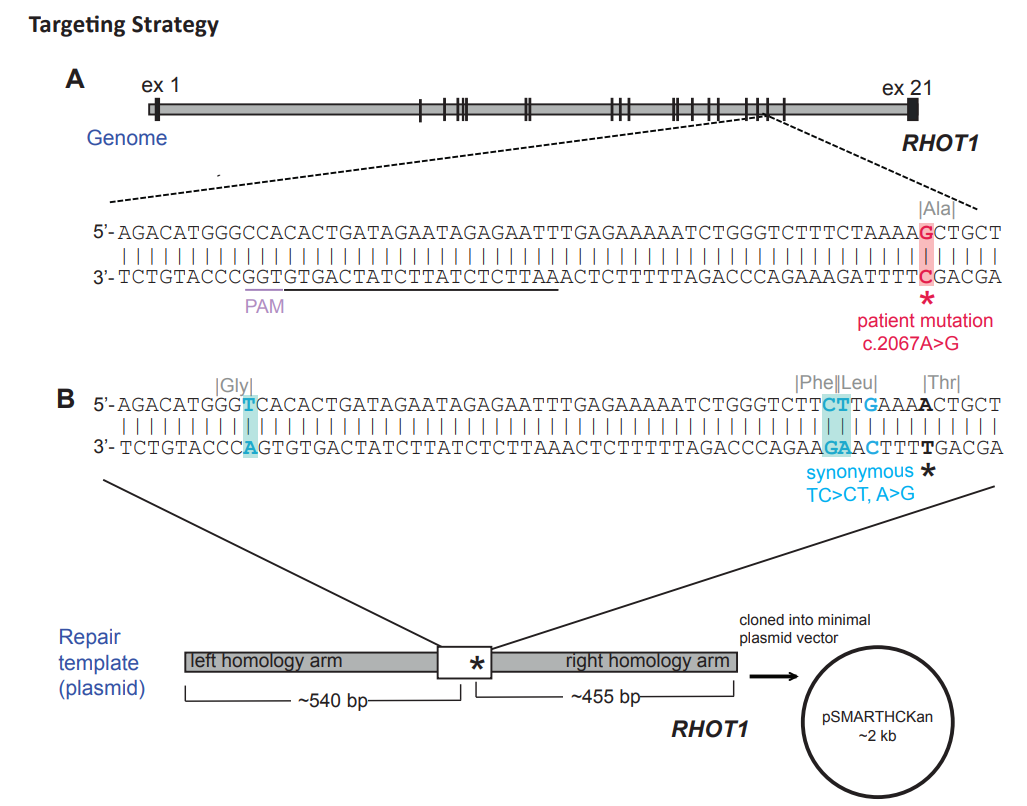 |
| --- |
| 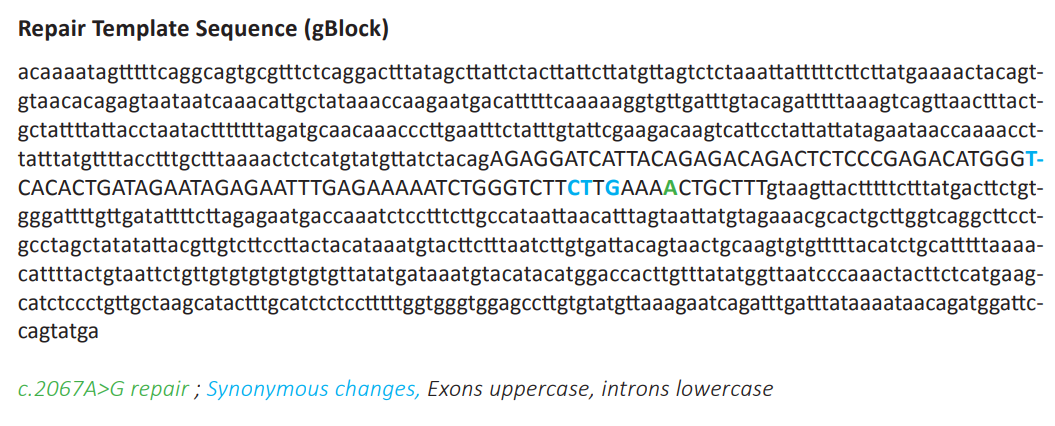 |
| **Supplementary Figure 2:** Schematic diagram of the targeting strategy for correction of c.2067A>G (p.T610A) mutation in exon 19 of RHOT1. (A) RHOT1 locus showing location of the patient-specific mutation (highlighted in red) and the Cas9-target site (protospacer). (B) Schematic diagram of the repair template (plasmid) used for homology directed repair comprising ~540 bp and 455 bp homology arms flanking the target site. Synonymous changes (highlighted in blue) have been introduced to facilitate the identification of correctly edited clones and to prevent Cas9-mediated re-cutting. |

| 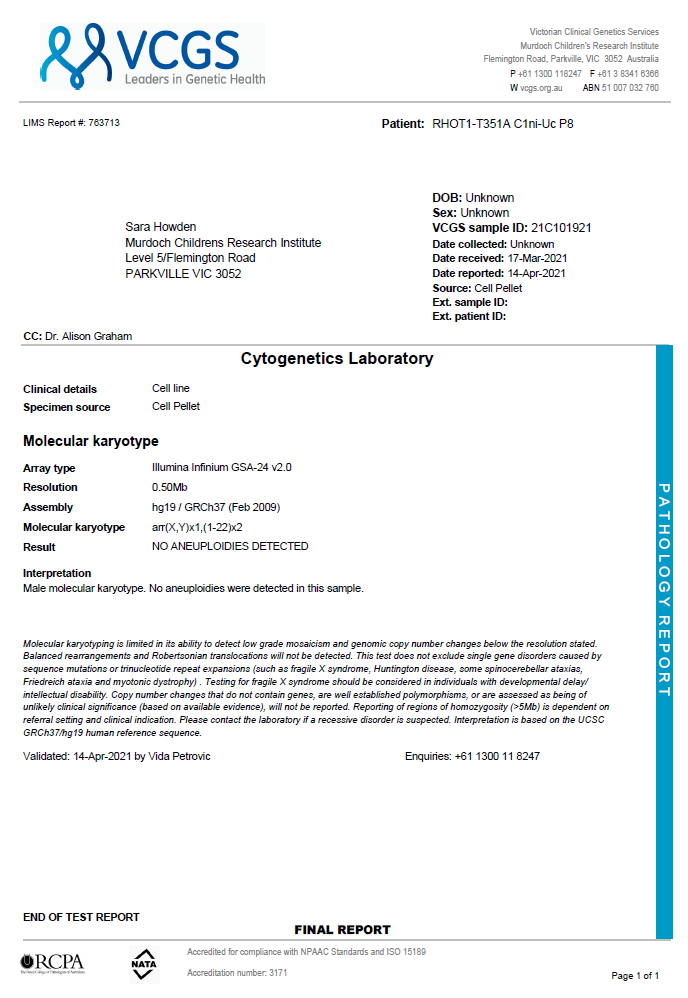 |
| --- |
| 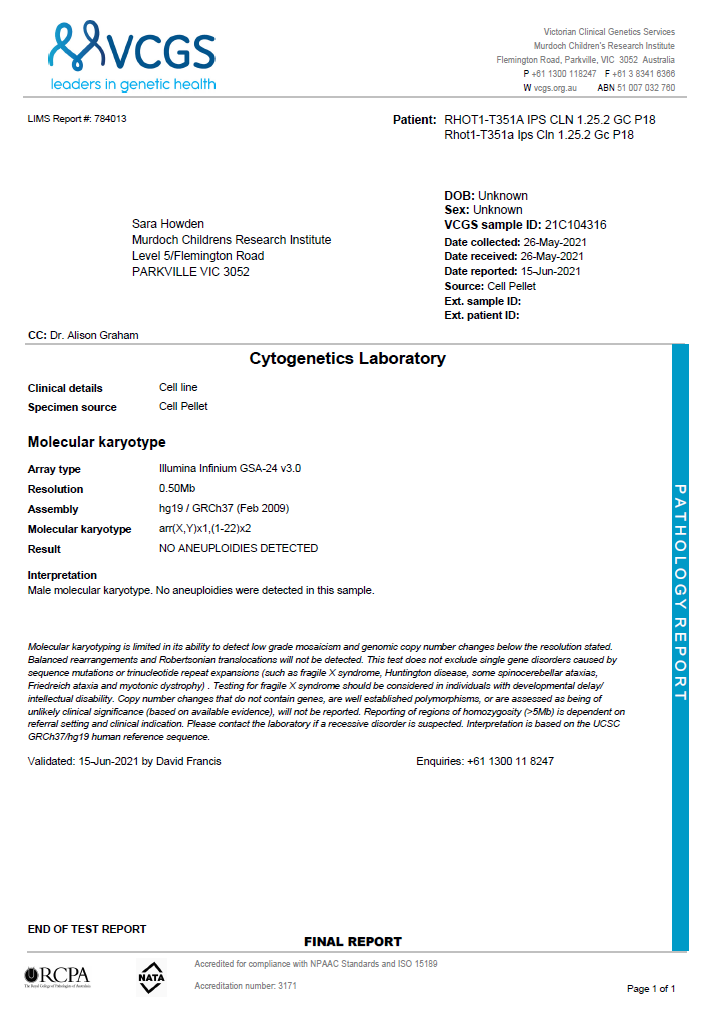 |
| 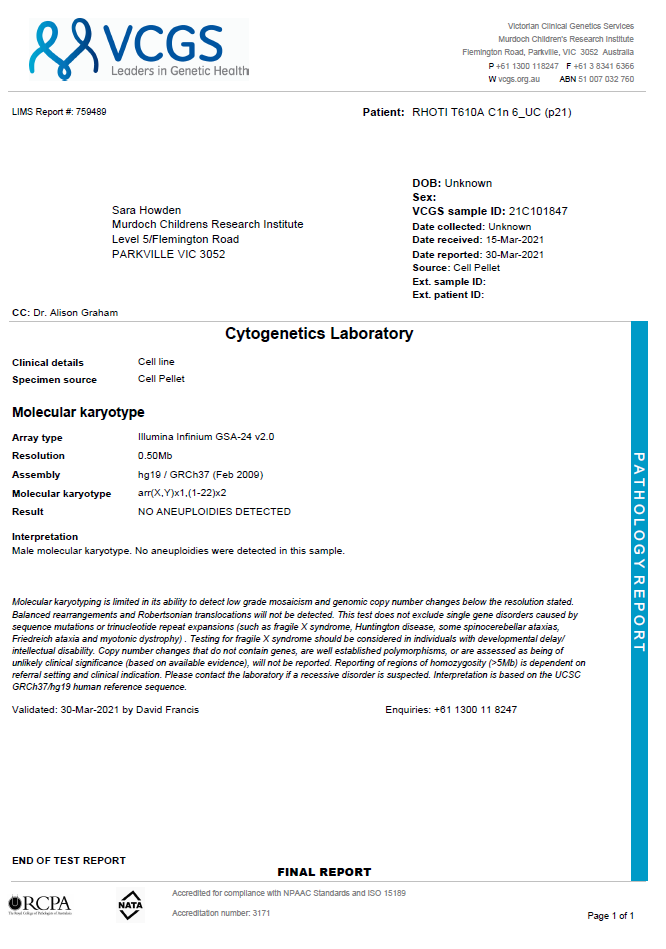 |
| 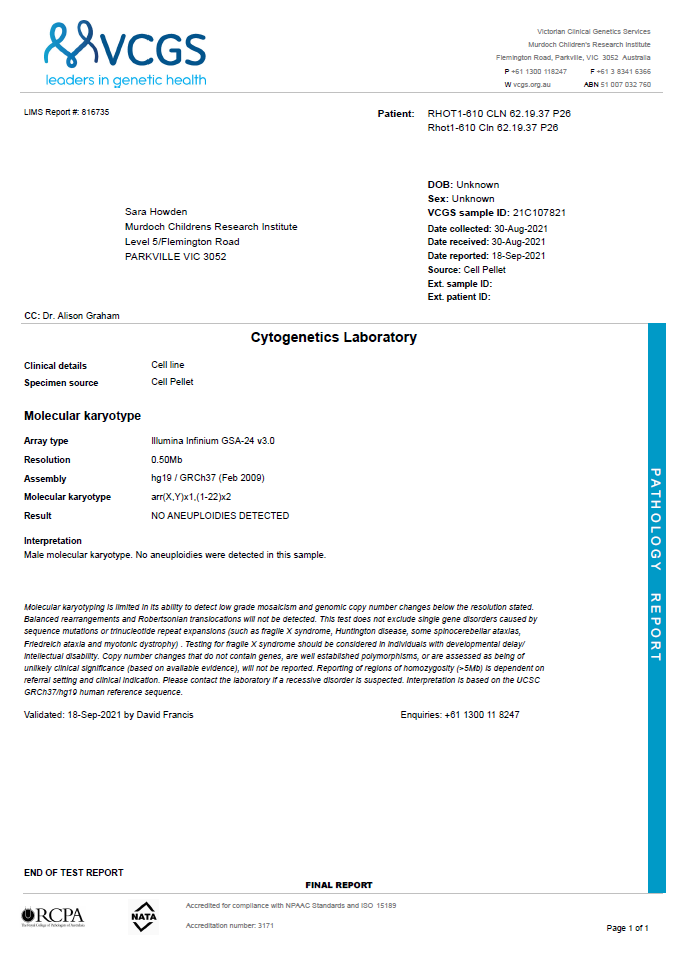 |
| **Supplementary Figure 3:** Patient-derived (RHOT1 T351A clone 1 and RHOT1 T610A clone 6) and gene edited (RHOT1 T351A-GC clone 25.2 and RHOT1 T610A-GC clone 62.19.37) iPSCs were submitted to VCGS for karyotype analysis by Illumina Infinium CoreExome-24 v1.1 SNP array. No aneuploidies were detected. |

| 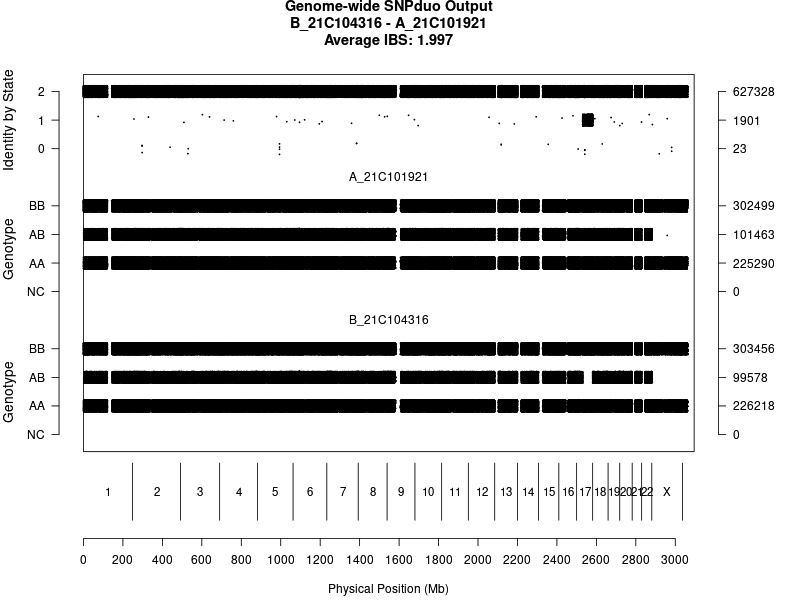 |
| --- |
| 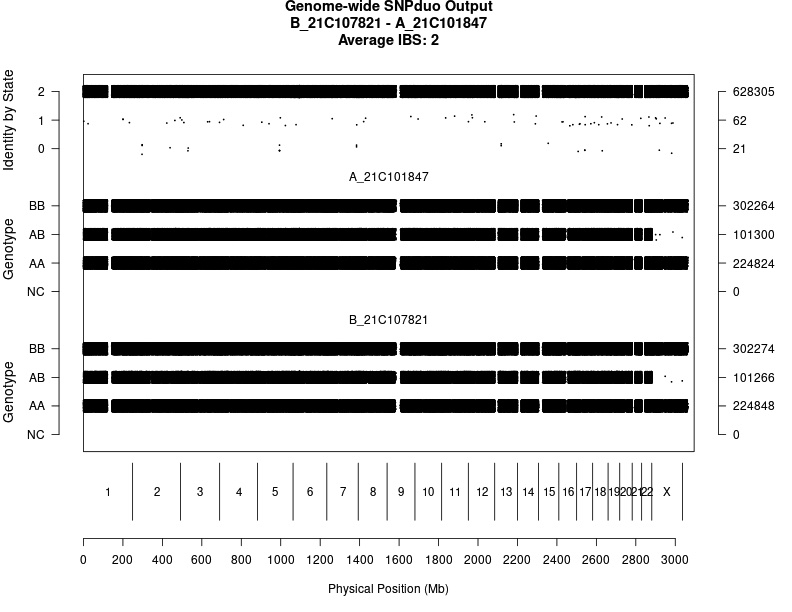 |
| **Supplementary Figure 4:** SNP-duo comparative analysis of the microarray genotyping data. Each SNP probe is plotted as a dot. Identity by state (IBS) plot shows the level of allele matching between two samples. IBS = 2, both alleles are identical; IBS = 1, one allele is shared; IBS = 0, no allele is shared. Results indicate identical SNP-genotypes for the entire genome between patient-derived (RHOT1 T351A clone 1 and RHOT1 T610A clone 6) and gene edited (RHOT1 T351A-GC clone 25.2 and RHOT1 T610A-GC clone 62.19.37) iPSCs. |

| \| 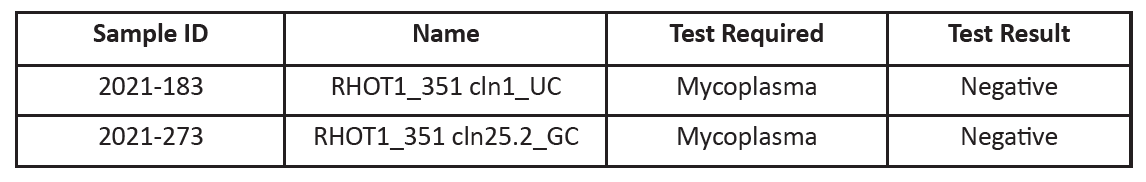 \| \| --- \| \| 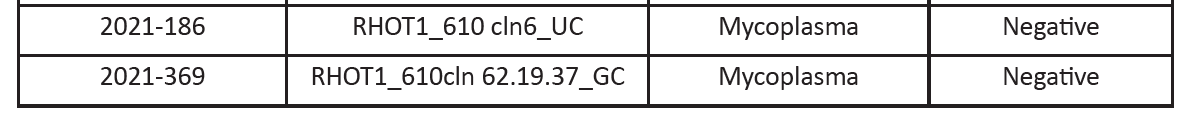 \| \| **Supplementary Figure 5:** iPSCs were submitted to Cerberus Sciences for mycoplasma testing and confirmed negative. \| |
| --- | --- | --- | --- |
|  |
